# Assessing the predictive efficacy of noninvasive liver fibrosis indices and portal vein diameter in predicting esophageal variceal bleeding in patients with cirrhosis

**DOI:** 10.1186/s13019-024-03047-5

**Published:** 2024-09-18

**Authors:** Xiaoxiao Lin, Qiaoli Lan, Ya Liu, Xiuli Dong, Lecan Wu

**Affiliations:** 1 Department of Gastroenterology, The Wenzhou Third Clinical Institute Affiliated to Wenzhou Medical University, Wenzhou People’s Hospital, No.57 Canghou Street, Wenzhou, Zhejiang 325000 China; 2 Department of Gastroenterology, The First Affiliated Hospital of Wenzhou Medical University, Wenzhou, Zhejiang 325000 China

**Keywords:** Esophageal varices bleeding, Liver fibrosis, Liver fibrosis indexes, Portal vein diameter, Liver cirrhosis

## Abstract

**Background:**

The objective of this study is to evaluate the diagnostic accuracy of noninvasive serum liver fibrosis markers and portal vein diameter (PVD) in predicting the occurrence of esophageal variceal bleeding (EVB) in patients with cirrhosis.

**Methods:**

A cohort comprising 102 individuals diagnosed with cirrhosis was divided into two groups: the P group (without EVB) and the PE group (with EVB). We conducted a comprehensive analysis comparing various noninvasive serum liver fibrosis indices, the Child-Pugh classification, ratios of aspartate aminotransferase to alanine aminotransferase, aspartate aminotransferase to platelet ratio index, fibrosis index based on four factors (FIB-4), PVD, and spleen thickness (SPT) between these groups. Receiver operating characteristic (ROC) curves were constructed for variables showing significant differences between the two groups, with subsequent calculation of the area under the ROC curve (AUROC) for each variable.

**Results:**

Significant distinctions were noted in the serum liver fibrosis markers between the P and PE groups, encompassing hyaluronic acid (HA), type III procollagen (PC-III), type IV collagen (IV-C), PVD, SPT, and FIB-4 (*p* < 0.05), as evidenced by univariate analysis findings. The respective AUROC values for these markers were 0.653, 0.706, 0.710, 0.730, 0.660, and 0.633. Additionally, upon integration with PVD, SPT, and FIB4, the AUROC values for liver fibrosis markers surged to 0.793, 0.763, and 0.706 correspondingly, highlighting the enhanced diagnostic potential.

**Conclusion:**

The integration of noninvasive liver fibrosis indices and PVD showcased remarkable diagnostic potential in EVB, underscoring its clinical relevance in predicting hemorrhagic events.

## Background

Esophageal variceal bleeding (EVB) is the most prevalent and hazardous complication associated with liver cirrhosis, affecting between 30 and 70% of individuals with this condition and posing a significant risk of mortality [1]. EVB serves as a critical indicator of the severity of liver cirrhosis, often accompanied by high rates of mortality and rebleeding. The 2016 consensus guidelines from the Asian Pacific Association for the Study of the Liver (APASL) highlighted the pivotal role of liver fibrosis in diagnosing and prognosticating liver cirrhosis, owing to its accuracy in gauging the extent of portal hypertension and the development of esophageal varices [2–4].

Recent studies and guidelines emphasize the importance of assessing portal hypertension (PH) through the measurement of hepatic venous pressure gradient, a valuable predictor of PH-related complications in cirrhotic patients [5]. However, due to its invasive nature, this technique is not routinely employed in clinical settings. Noninvasive methods for assessing liver fibrosis, such as measuring indices like hyaluronic acid (HA), type III procollagen (PC-III), and type IV collagen (IV-C), have gained prominence in clinical practice [6]. Although these indices have been shown to correlate with cirrhosis prognosis, their utility in predicting EVB is still not fully understood.

In another study, various markers including aspartate aminotransferase (AST) to alanine aminotransferase (ALT) ratio (AAR), AST to platelet ratio index (APRI), platelet count to spleen diameter (PC/SD), fibrosis-4-index (FIB-4), fibrosis index (FI), and King’s Score were evaluated for their predictive efficacy in esophageal variceal bleeding among Albanian patients diagnosed with liver cirrhosis. The findings indicated that FIB-4 emerged as the most effective noninvasive liver fibrosis marker, offering promise as an initial screening tool for cirrhotic patients [7].

In this study, we sought to investigate the potential and effectiveness of liver fibrosis markers in predicting EVB, thereby laying a groundwork for the prevention, management, and prognosis of EVB in individuals with cirrhosis.

## Materials and methods

### Materials

A retrospective analysis was conducted on 102 cirrhotic patients with esophageal varices who were consecutively admitted to our hospital between January 2011 and January 2022. This cohort consisted of 66 males and 36 females, with ages ranging from 28 to 82 years. Liver cirrhosis was attributed to various causes: hepatitis (*n* = 64), alcohol consumption (*n* = 20), cholestatic disorders (*n* = 2), and unknown etiology (*n* = 16). The diagnosis of liver cirrhosis was based on a comprehensive assessment that included patient history, physical examination, laboratory tests, ultrasound, abdominal computed tomography (CT) imaging, and liver biopsy [8]. Liver puncture was not the only criterion. Gastroscopy was employed to ascertain the presence and characteristics of esophageal varices, which were categorized based on size (small, medium, or large) and location, as per the Sarin classification (GOV: gastroesophageal varices; IGV: isolated gastric varices) [9]. All included patients underwent gastroscopy within one week of admission for definitive diagnosis.

The primary concern associated with esophageal varices is the risk of bleeding. Indications of bleeding esophageal varices encompass vomiting substantial amounts of blood, experiencing black, tarry, or bloody stools, feeling lightheaded due to blood loss, and in severe cases, losing consciousness. Exclusion criteria for this study encompassed patients with hepatocellular carcinoma, those who underwent endoscopic esophageal varix ligation, or individuals with severe organ dysfunction. The Ethics Committee of the Wenzhou People’s Hospital approved the study protocol.

### Methods

The study encompassed 102 patients with cirrhosis who were categorized into two groups: the P group (without EVB), and the PE group (with EVB). Various serum markers and other scores of liver fibrosis, such as HA, PC-III, IV-C, Child-Pugh stage, AAR, APRI, FIB-4, portal vein diameter (PVD), and spleen thickness (SPT), were retrospectively examined. Blood samples were taken within 24 h after admission. Serum levels of HA, PC-III, IV-C, Child-Pugh stage, AAR, APRI, FIB-4 were measured by commercially available kits purchased from Shenzhen Yahui Long Biotechnology Co., Ltd. (China) using chemiluminescence assay. PVD and SPT were measured using ultrasound. The patient was positioned in supine position, and the internal diameter of the main portal vein was measured from the body surface using an ultrasound probe. In the right lateral position, the probe was placed between the anterior and posterior axillary lines to obtain the thickness of the spleen. The examination was completed during the patient’s hospitalization.

Through univariate analysis, variables exhibiting significant differences between the two groups were identified. Subsequently, the receiver operating characteristic (ROC) curve and area under the ROC (AUROC) values were computed to assess the diagnostic efficacy of these variables in predicting esophageal variceal bleeding. Significant variables were further analyzed using binary logistic regression to establish their combined predictive value. Adherence to the Declaration of Helsinki principles was ensured throughout the study, and the Ethics Committee of Wenzhou People’s Hospital approved the research protocol (KY02022-027).

### Statistical analysis

SPSS 22.0 software was utilized for statistical analysis. For normally distributed measurement data, mean ± standard deviation (SD) were presented and compared using the *t*-test. Skewed variables were expressed as median and interquartile range (IQR), with differences assessed via nonparametric tests. Enumeration data were compared using the chi-squared (χ2) test, while grade data were analyzed using the rank-sum test. Significance was set at *p* < 0.05. Variables exhibiting significant differences between groups were identified. To evaluate diagnostic performance, ROC analysis was employed for these variables, with each AUROC value calculated. Binary logistic regression was then applied to amalgamate these variables. Subsequently, the ROC curve was constructed, and AUROC was computed based on the amalgamated variables.

## Results

Univariate analysis between the two groups.

Univariate analysis revealed notable differences in noninvasive serum liver fibrosis markers, such as HA, PC-III, IV-C, PVD, SPT, and FIB-4, between the P and PE groups (*p* < 0.05) (Table 1).

**Table 1:** Univariate analysis of patient characteristics and factors associated with EVB between the two groups

Variables	PE group (*n* = 42)	P group (*n* = 60)	*P* value
Gender (M/F)	28/14	38/22	0.729
Age(year), mean ± SD	55.93 ± 10.98	58.33 ± 11.96	0.304
HA (µg), median (IQR)	537.05(628.67)	316.51(375.21)	0.009
PC-III(µg), median (IQR)	133.45(124.85)	62.30(76.98)	0.000
IV-C(µg), median (IQR)	103.39(64.07)	61.30(52.55)	0.006
ALT(U/L), median (IQR)	32(13.75)	31(22.5)	0.496
AST(U/L), median (IQR)	40(15.5)	46(28.25)	0.051
PLT count (10^9^/L), median (IQR)	53.5(55.25)	88.5(77)	0.000
AAR, median (IQR)	1.38(0.82)	1.52(0.82)	0.671
APRI, median (IQR)	1.79(1.55)	1.39(1.71)	0.242
FIB4,median (IQR)	4.05(3.91)	3.43(3.99)	0.023
Child-Puge classification, n(%)			0.213
A	12(28.6%)	27(45%)	
B	22(52.4%)	26(43.3%)	
C	8(19%)	7(11.7)	
PVD(mm), median (IQR)	12(2.25)	11(2)	0.000
SPT(mm), median (IQR)	48(11)	42(8)	0.006

ROC curves of the factors with significant differences associated with EVB between the two groups.

**Fig. 1:**
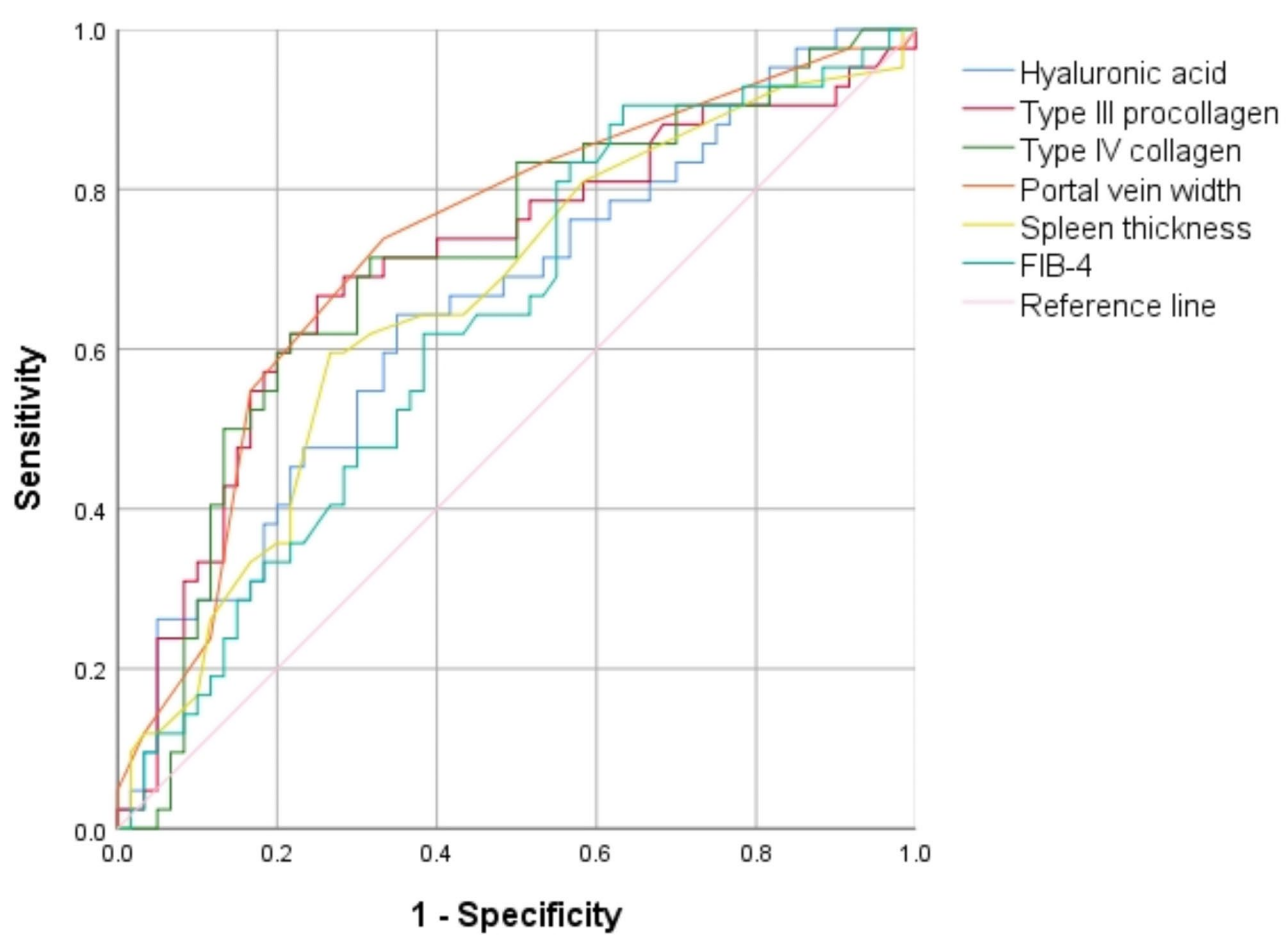
Performance of serum liver fibrosis indexes, PVD, SPT, and FIB-4 in the detection of EVB. ROC curves demonstrate the diagnostic accuracy of HA, PC-III, IV-C, PVD, SPT, and FIB-4 in predicting the presence of EVB in patients with liver cirrhosis

The ROC curves were generated for several liver fibrosis indices including HA, PC-III, IV-C, PVD, SPT, and FIB-4, demonstrating their corresponding AUROC values of 0.653, 0.706, 0.710, 0.730, 0.660, and 0.633. Notably, these results underscore the robust diagnostic capabilities of these indices, particularly highlighting the efficacy of PVD in conjunction with other liver fibrosis markers (Fig. 1; Table 2).

**Table 2:** The ROC curve values of the factors with significant differences associated with EVB between the two groups

	95% CI	Sensitivity	Specificity
HA	0.545–0.761	0.643	0.600
PC-III	0.599–0.813	0.714	0.667
IV-C	0.606–0.814	0.714	0.683
PVD	0.629–0.831	0.548	0.833
SPT	0.551–0.768	0.405	0.783
FIB-4	0.525–0.741	0.619	0.617

CI, confidence interval of AUC values

Diagnostic performance of combined indicators.

**Fig. 2:**
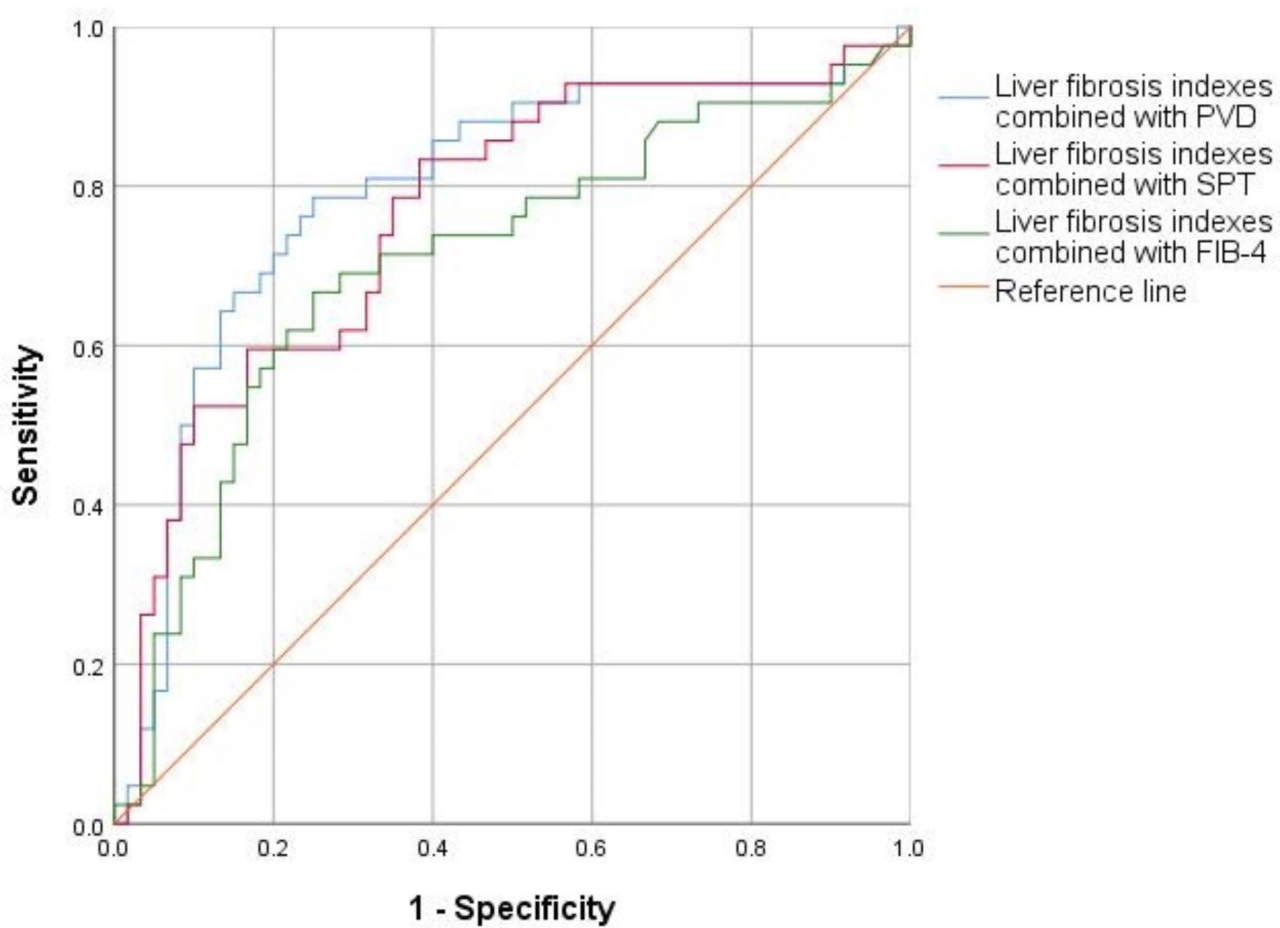
Performance of combined variables in the detection of EVB. ROC curves demonstrate that a combination of serum liver fibrosis indices and PVD result in higher diagnostic accuracy in predicting the presence of EVB in patients with liver cirrhosis

We incorporated liver fibrosis markers (HA, PC-III, and IV-C) in conjunction with PVD into a binary logistic regression model to estimate prediction probability (PRE_1). Subsequently, the ROC curve was generated, yielding an AUROC of 0.793. Following this, PRE_2 was calculated by incorporating liver fibrosis markers and SPT using binary logistic regression, with resulting ROC curves and an AUROC of 0.763. Lastly, the diagnostic accuracy of liver fibrosis markers in conjunction with FIB-4 was evaluated, revealing an AUROC of 0.706 (refer to Fig. 2; Table 3). Consequently, the diagnostic efficacy of liver fibrosis markers combined with PVD surpasses that of their combination with SPT or FIB-4, and outperforms individual variables in EVB among cirrhotic patients.

**Table 3:** Diagnostic performances of combined indicators

	95% CI	Sensitivity	Specificity
Liver fibrosis indexes combined with PVD	0.699–0.888	0.683	0.786
Liver fibrosis indexes combined with SPT	0.666–0.860	0.667	0.738
Liver fibrosis indexes combined with FIB-4	0.599–0.813	0.633	0.714

CI, confidence interval of AUC values

## Discussion

EVB constitutes a common and severe complication often encountered in patients with liver cirrhosis. EVB manifests with sudden onset, rapid deterioration, and a significantly heightened fatality rate. Despite its gravity, there exists a conspicuous absence of effective predictive models or systems for assessing EVB risk in clinical settings. In recent years, there has been a surge of interest in noninvasive index prediction of EVB within academic circles [10]. In this study, we conducted a comparison among various noninvasive serum liver fibrosis indicators, including but not limited to HA, PC-III, IV-C, Child-Pugh stage, AAR, APRI, FIB-4, PVD, and SPT.

Patients diagnosed with cirrhosis were categorized into two groups, namely the P group (without EVB) and the PE group (with EVB). Presently, a variety of methods are employed to assess the risk and prognosis of EVB, with noninvasive markers being particularly emphasized [11, 12]. In this study, a univariate analysis was undertaken, revealing notable differences in HA, PC-III, IV-C, PVD, SPT, and FIB-4 between the aforementioned groups. Recent studies have demonstrated that the stage of liver cirrhosis, when combined with hepatic venous pressure gradient, may predict the likelihood of EVB [13]. Nonetheless, invasive procedures are often poorly tolerated by patients and are not universally applicable. Employing binary logistic regression, we integrated these variables, constructed the ROC curves, and computed the AUROC. Our findings indicate that a composite of liver fibrosis indicators, comprising HA, PC-III, IV-C, and PVD, exhibits the highest diagnostic efficacy for EVB.

Liver fibrosis, a pathological state, manifests through an abnormal accumulation of extracellular matrix elements within the liver, potentially progressing into decompensated cirrhosis if left unaddressed, leading to severe health ramifications. In contemporary medical practice, indicators like HA, PC-III, and IV-C are routinely utilized to gauge cirrhosis severity.

Extensive research highlights the exceptional effectiveness of HA in precisely determining the extent of liver cirrhosis [14, 15]. Elevated concentrations of HA are consistently observed during both the active and advanced stages of liver disease, highlighting its potential close association with the progression of liver fibrosis. This is evidenced by the positive correlation between HA levels and the severity of fibrosis [16].

PC-III, derived from both hepatocytes and interstitial cells, is present in the bloodstream. Evaluating serum PC-III levels provides insight on the synthesis of collagen by hepatocytes. As an amino-terminal polypeptide, PC-III acts as a precursor to type III collagen, which is prevalent in the early phases of liver fibrosis. Thus, monitoring PC-III provides critical insights into the metabolism of type III collagen during these early fibrotic processes. Research findings indicate that PC-III is a promising metric for the assessment of liver fibrosis [17, 18].

IV-C, a key component of the hepatic basement membrane, exhibits escalated synthesis subsequent to liver injury, potentially functioning as a marker for the severity of liver fibrosis. Previous studies have revealed a significant positive correlation between serum levels of HA and IV-C among patients, along with the extent of esophageal varices [19]. Prior investigations have demonstrated a direct correlation between HA levels and the severity of esophageal varices [20, 21].

Hence, the elevation in serum levels of these markers likely mirrors the advancement of liver cirrhosis and the extent of esophageal varices, indicative of a heightened risk of bleeding commensurate with the severity of the varices. Moreover, in this study, no significant variances in age or Child-Pugh classification were discerned between the two groups.

Prior studies have scrutinized the risk factors for EVB in cirrhotic patients. Findings suggest that factors such as gender and age exhibit minimal correlation with EVB, while a higher Child-Pugh score demonstrates a notable association with its incidence. However, it is important to note that, despite its correlation with the incidence of EVB in cirrhosis, the Child-Pugh classification does not constitute an independent risk factor and possesses limited clinical significance in the prognostic assessment of EVB [22].

PH is a prominent clinical manifestation of liver cirrhosis, significantly impacting mortality rates among afflicted individuals [23]. PVD acts as an indirect gauge of PH severity, with higher PVD levels correlating with more pronounced EV and heightened susceptibility to EVB. Measurements obtained through real-time shear wave elastography in patients with cirrhosis have shown a direct relationship between PVD and the severity of EV, indicating that an increase in PVD is associated with more severe cases of EV. Enhanced MRI has demonstrated potential in predicting PH and identifying high-risk EV in patients with cirrhosis due to hepatitis B, highlighting the association between PV characteristics and EVB risk. Integrating MRI with PVD measurement, as evidenced in studies conducted in China, offers a comprehensive approach for assessing severe EV, demonstrating high diagnostic efficacy. Numerous studies have independently verified PVD as a significant risk factor for EVB and subsequent rebleeding events.

## Conclusion

Compared with the individual predictive risk, the integration of serum liver fibrosis markers and PVD enhances the effectiveness of predicting the onset of EVB and plays a substantial role in markedly improving the disease prognosis in clinical settings. In addition, the laboratory indicators and imaging examinations involved in this study are based on the premise of non-invasive examination. Compared with invasive examination, they are more acceptable to patients and can be widely used in clinical practice in the future.

## Limitations

The aim of this study was to explore the efficacy of combined indicators in a non-invasive approach for predicting EVB occurrences in clinical settings. It is crucial to acknowledge that this study was carried out in a single center with a restricted sample size. Consequently, it is imperative for forthcoming studies to prioritize multi-center trials to bolster the applicability of the results. Particularly, there is a need for further scrutiny into the training and validation sets.

## Data Availability

The datasets used and/or analysed during the current study available from the corresponding author on reasonable request.

## Abbreviations

EVB: Esophageal varices bleeding
PVD: Portal vein width
EV: Esophogeal varices
AST: Aspartate aminotransferase
ALT: Alanine aminotransferase
AAR: AST-to-ALT
APRI: AST-to-platelet ratio index
FIB-4: Fibrosis index based on the four factors
PVD: Portal vein width
SPT: Spleen thickness
ROC curve: Receiver Operating Characteristic curve
AUROC: Area under the ROC curve
HA: Hyaluronic acid
PC-III: Type III procollagen
IV-C: Type IV collagen
APASL: Asian- Pacific Association for the Study of the Liver
HVPG: Hepatic venous pressure gradient
PH: Portal hypertension
